# Considering land tenure in REDD+ participatory measurement, reporting, and verification: A case study from Indonesia

**DOI:** 10.1371/journal.pone.0167943

**Published:** 2017-04-13

**Authors:** Mary Elizabeth Felker, Indah Waty Bong, Walker Holton DePuy, Lina Farida Jihadah

**Affiliations:** Center for International Forestry Research, Bogor, Indonesia; University of Waterloo, CANADA

## Abstract

Measurement, Reporting, and Verification (MRV) systems are thought to be essential for effective carbon accounting and joint REDD+ carbon, conservation, and social development goals. Community participation in MRV (PMRV) has been shown to be both cost effective and accurate, as well as a method to potentially advance stakeholder empowerment and perceptions of legitimacy. Recognizing land tenure as a long-standing point of tension in REDD+ planning, we argue that its engagement also has a key role to play in developing a legitimate PMRV. Using household surveys, key informant interviews, and participatory mapping exercises, we present three ‘lived’ land tenure contexts in Indonesia to highlight their socially and ecologically situated natures and to consider the role of tenure pluralism in shaping PMRV. We then raise and interrogate three questions for incorporating lived land tenure contexts into a legitimate PMRV system: 1) Who holds the right to conduct PMRV activities?; 2) How are the impacts of PMRV differentially distributed within local communities?; and 3) What is the relationship between tenure security and motivation to participate in PMRV? We conclude with implementation lessons for REDD+ practitioners, including the benefits of collaborative practices, and point to critical areas for further research.

## Introduction

Current international efforts to mitigate climate change include actions targeting the sustainable conservation and management of tropical forested landscapes. First proposed at the UN Framework Convention on Climate Change (UNFCCC) in 2005, Reducing Emissions from Deforestation and Forest Degradation (REDD+) policy aims to compensate developing countries for their avoided carbon emissions through reduced deforestation and forest degradation in addition to the conservation, sustainable management, and enhancement of forest carbon stocks [1].

As currently envisioned, REDD+ requires a standardized unit of carbon, representing reduced carbon emissions, to be calculated, monitored, and monetized for global exchange [2,3]. Measurement, Reporting, and Verification (MRV) systems are thought to be essential for both effective carbon accounting and joint carbon, conservation, and social development ‘REDD+ Readiness’ goals [3,4]. Critics of forest carbon accounting argue that the processes of simplification and standardization required in MRV risk minimizing the priority of non-carbon forest benefits, such as biodiversity conservation and local livelihoods, and excluding alternative forest knowledge and values [2,5–9]. Carbon accounting has likewise been linked to the possible recentralization of forest management and the disempowering of local communities [6].

Together such concerns led to the inclusion of social and environmental ‘safeguards’ in the 2010 UNFCCC Cancun Agreements. In policy and planning circles, community-centered safeguards have focused on questions of tenure security, stakeholder participation, and the need for free, prior, and informed consent (FPIC) [10,11]. Tenure security is broadly defined as when resource rights are respected and upheld across both de jure and de facto realms [12], while FPIC ensures REDD+ projects result from not merely previously held consultations, but fully informed, non-coercive, and participatory community agreements [11]. Recognizing the importance of stakeholder participation, the Permanent Forum on Indigenous Peoples has argued for indigenous and forest-dwelling peoples’ involvement in the design and implementation of MRV processes [13]. Grounded in the “idiom of legitimacy, fairness and rights” ([14] p.3545), such social safeguards aim to place questions of indigenous rights and participation at the forefront of international conservation projects [15–17].

Research on participatory MRV (PMRV) has shown community involvement to be both cost effective and accurate [4,18], as well as linked to positive empowerment outcomes and activity sustainability [19]. Gupta, et al. [3], however, argue that MRV design and implementation should place questions of accountability and legitimacy at their center, in particular focusing on “*by and for whom forests are taken into account*” (p.727; emphasis in original).

The rise of global environmental governance showcases the growing role of non-state actors, whether multi-national companies, non-governmental organizations, or indigenous communities, in determining the effectiveness of environmental mechanisms and institutions [20,21]. From in depth community-based conservation research to emerging UNFCCC safeguard policies, local stakeholder perceptions of legitimacy are increasingly seen as critical for the success of conservation projects and REDD+ programs particularly [20–22]. In the context of forest carbon accounting, Gupta, et al. argue that a “legitimate” MRV ([3] p.729) includes respecting and engaging local forest knowledge in MRV design and implementation; attention to local values and local needs; and being responsive to cultural context and diversity. PMRV research suggests that legitimacy can be advanced by integrating MRV practices into local contexts [19,23].

Land tenure in tropical forested countries has been highlighted as a driving factor limiting REDD+ outcomes [12,24,25]. Attention to “local framings” of land tenure has likewise been posited as critical for designing both equitable and effective REDD+ programs ([26]p.201). While select studies advance the potential for PMRV activities to improve local peoples’ access to land and resources [19,27,28], we argue that there is still a need for nuanced analysis of how local land tenure contexts might interact with PMRV design and in particular the implementation of a legitimate PMRV.

Specifically, we argue for more attention by REDD+ practitioners and policymakers to how legal pluralism manifests on the ground [29], especially across the heterogeneous landscapes of Southeast Asia [26]. Legal pluralism sees the law not as a singular, state-based institution, but instead a constellation of legal systems operating across local, national, and international scales [30–32]. Stepping away from a State-dominated legal understanding is thought to allow for a more accurate description of how individuals and groups engage with and across both diverse stakeholders (such as multi-national corporations, non-governmental organizations, nation states, and indigenous communities [33]) and legal systems (those statutory and customary), as well as how those legal systems interact [30,34,35].

Indonesia, with its diversity of socio-ecological contexts, co-existing legal systems, and minimal statutory recognition of community rights [34–36] provides an especially valuable context to consider the role of land tenure pluralism in shaping a participatory MRV system. With this in mind, our results present three ‘lived’ land tenure contexts in Indonesia to highlight their socially and ecologically situated natures and examine how land tenure is experienced and negotiated in particular places.

**Fig 1:**
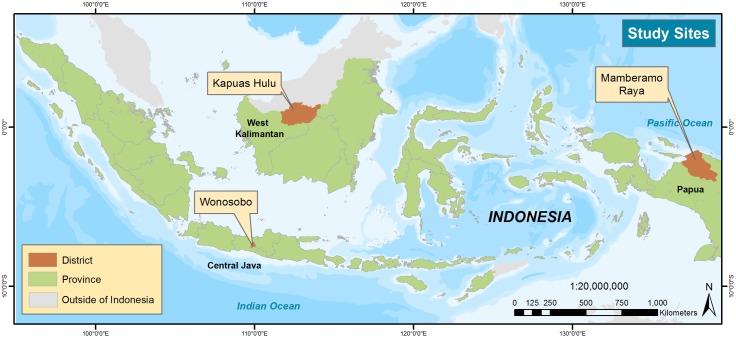
Map of Study Site Locations.

We begin this paper with a short review of forest governance in Indonesia, a presentation of our methods, and a description of our study sites. We then present results depicting three lived tenure contexts seen in the Indonesian provinces of Central Java, West Kalimantan, and Papua (Fig 1). The following discussion focuses on how these contexts illuminate particular opportunities and challenges for PMRV. These include questions regarding the right to conduct PMRV activities, the differential impact(s) of PMRV for local communities, and potential motivations to participate in PMRV. We conclude by considering the gains made through such investigations towards a *legitimate* PMRV.

## Forest governance in Indonesia

Legal pluralism in Indonesia stretches from the colonial period to the transnational governance regimes of today [37]. In Indonesia, this pluralism can be seen across provinces, where the State’s statutory legal system and various sub-national customary and statutory systems are seen to “interact and co-exist” rather than operate in opposition to one another ([16]p.129]). In practice, legal pluralism has had a dynamic history in Indonesia, with the authority of statutory and customary systems shifting over time, across scales of governance, and to varying extents throughout the country. The following section briefly outlines the history of forest governance in Indonesia as it relates both to legal pluralism and the position of local communities within the statutory legal system.

State law, as established by Dutch colonial administrators, explicitly differentiated between systems of ‘colonial law’ and the ‘people’s law,’ which included customary (*adat)* and religious systems as practiced throughout Indonesia. In 1945, the newly independent Indonesian government then replaced colonial law with ‘national law,’ which sought to incorporate these systems of customary and religious law into a single legal system [38]. Further national laws established during President Suharto’s administration, beginning in 1966, limited the recognition of customary law. The farthest-reaching of these laws was the Forestry Act of 1967 (Forestry Act, Act No. 5 of 1967 now subsumed under the New Forestry Act No 41 of 1999), which provided the Ministry of Forestry with tenure rights to all of Indonesia’s forest lands. The law states that in the National Forest Estate customary law should only be recognized when it does not oppose national and state interests. Contemporary scholars recognize that this law stripped Indonesian communities living in and around forests–approximately 100 million people–of legal rights to their lands [36].

The National Forest Estate is divided into three main zones: Conservation Forest, Protection Forest, and Production Forest. These classifications, which delineate the activities permitted within each, were made without consideration for previous land claims or vegetation cover [39]. Conservation Forest allows for research, education, and limited tourism; Protection Forest for the collection of Non-Timber Forest Products (NTFPs); and Production Forest for commercial timber extraction.

The Reformation Period, following Suharto’s resignation in 1997, resulted in a notable shift from a highly centralized government to a more decentralized one, devolving certain management rights to district governments (Law No. 22, 1999 and reformed under Law No. 32, 2004). District governments thus gained the right to manage Non-Forest Estate land (Law No 41, 1999), though the Forest Estate remained under the Ministry of Forestry’s purview [40]. District regulations in some areas increased community recognition and land rights.

At the provincial level, there was a separate push towards decentralization with the granting of special autonomy status to the provinces of Aceh and Papua in 2001 [41]. This status devolves powers to the provincial government to create, implement, and enforce its own laws and retain a larger percentage of the tax on natural resource extraction (Article 38 and 42). Despite a higher recognition of customary institutions and laws, however, both national and regional statutory governments still hold substantial authority [38]. Throughout the country, decentralization remains incomplete, with most local decisions able to be overruled by the national government [36]. This mixed devolution of control has led to unclear governance roles, tenure rights, and claims to authority [42,43].

Though decentralization has led to greater empowerment of community management regimes, these rights are narrowly supported in national statutory law. Local communities living within or near National Forest Estates often find their customary (*adat*) legal systems disenfranchised and their options for claiming additional rights limited [38]. National statutory law, however, does contain certain avenues for local communities to gain partial recognition of forest rights. Two such community forestry programs pertinent to our research are the Forest Partnership program (*Hutan Kemitraan*) (Basic Forestry Law No 41/1999 and Government Regulation No 6/2007 Article 99) and the Village Forest program (*Hutan Desa*) (Basic Forestry Law No 41/1999 and Government Regulation No 6/2007 Article 99). Forest Partnerships provide rights to local communities on a case-by-case basis according to a formal agreement between a private land holder and a community [44]. The Village Forest program, in contrast, provides village-based institutions the right to manage and protect a determined area of forest under a 35-year lease [36].

Recent changes in forest governance at the national level highlight the fluidity of legal pluralism in Indonesia. In a promising advancement for community rights recognition, the Indonesian Constitutional Court ruled in 2013 that the classification of Indigenous Community Forests (*Hutan Masyarakat Adat*) within the National Forest Estate is unconstitutional (Customary Court Case 35/PUU-X/2012). By removing these lands from the National Forest Estate, the decision provides indigenous communities with full rights to these forest areas. Though it has yet to be implemented on the ground, this is the first time the Indonesian government has devolved full land rights to communities [35].

This brief history of forest governance in Indonesia illustrates the multiple, simultaneous tenure systems that have shaped people’s relationships to forests in the past and those continuing today. Understanding this dynamic history and the mixed devolution of statutory rights seen across Indonesia is necessary to fully appreciate and interrogate the local framing of land tenure contexts presented in Section Three.

### Site selection and methods

The results presented in this paper are part of a larger research project on Participatory Measurement, Reporting, and Verification (PMRV) conducted by researchers at the Center for International Forestry Research (CIFOR) [45]. The study focused on three Indonesian provinces that represent a gradient of forest states and a diversity of governance arrangements and local livelihoods. Our research sites (Fig 1) included two villages in Wonosobo district, Central Java (Lebak and Karanganyar), three in Kapuas Hulu district, West Kalimantan (Hulu Pengkadan, Sri Wangi, and Nanga Jemah), and two in Mamberamo Raya district Papua (Bagusa and Yoke). Research site typologies are outlined in Table 1 according to data from the Indonesian Provincial Central Statistics Agencies of Central Java Province [46], West Kalimantan Province [47], and Papua Province [48] and the Indonesian Ministry of Forestry [49]. Statutory classifications have corresponding use rights described in the Forest Governance in Indonesia section above. The primary data was collected by a multidisciplinary team, including the authors, between July 2013 and January 2014.

**Table 1:** Site Typologies.

Province	Central Java	West Kalimantan	Papua
District	Wonosobo	Kapuas Hulu	Mamberamo Raya
Study villages	• Lebak • Karanganyar	• Hulu Pengkadan • Nanga Jemah • Sriwangi	• Yoke • Bagusa
**Demographic (district)**^**1**^			
Area size (km^2^)	985	29,842	28,035
Population density (people/km^2^)	789	8	0.77
**Sampled households (village)**^**2**^			
Number of households in village (# of sampled households)	• Lebak: 397 (81) • Karanganyar: 686 (90)	• Hulu Pengkadan: 191 (64) • Nanga Jemah: 201 (65) • Sriwangi: 133 (56)	• Yoke: 54 (28) • Bagusa: 57 (34)
**Socio-economic conditions (village)**^**3**^			
Type of community	Javanese	Malay/Dayak	Yoke and Bagusa Papuan
Main livelihoods	• Farming • Harvesting and selling timber and other non-timber forest products from plantation forest • Wage labor • Livestock and fish farming	• Shifting cultivation • Tapping rubber • Harvesting timber and other non-timber forest products • Artisanal gold mining	• Fishing • Harvesting timber and other non-timber forest products • Agroforestry • Sago gardening • Hunting
Economic pressure (presence of private sector in the studied villages)	• Long-established state-owned forest plantation company (Perhutani)	• Historical logging concession (inactive at present but still holds timber use rights)	• Historical oil exploration • Ongoing logging concession
**Forest conditions (village)**^**4**^			
Forest cover	• LOW • Predominately planted forest	• MEDIUM • Predominately secondary forest, logged over forest, and natural forest	• HIGH • Predominately natural forest, secondary forest, and logged over forest
Forest Estate regime	• Limited Production Forest • Permanent Production Forest	• Protection Forest ** • Limited Production Forest	• Conservation Forest Limited Production Forest* • Permanent Production Forest* • Convertible/Conversion Production Forest*
Active land use permit	• Forest Partnership PHBM	• Timber Utilization Permit in Natural Forest (IUPHHK-HA) PT. Harapan Kita Utama*** • Village Forest (in progress)**	• Timber Utilization Permit in Natural Forest (IUPHHK-HA) PT Mamberamo Alas Mandiri*

^**1**^ Indonesian Provincial Central Statistics Agencies, 201.

^**2**^ Number of households in each village derived from the village demographic book and adjusted to our definition of a household as a group of people living under the same roof and pooling resources (labor and income).

^**3**^ HHS and KII, this study.

^**4**^ Developed from Indonesian Ministry of Forestry data.

* Located only in Bagusa, Papua.

** Located in Nanga Jemah and Sri Wangi villages, West Kalimantan.

*** Located in Nanga Jemah village, West Kalimantan.

For this paper, a common set of research methods was designed to examine land tenure contexts shared across research site villages, as well as variations between them. The methods include focus group discussions (FGD), key informant interviews (KII), and household surveys (HHS).

### Participatory mapping focus group discussion

For each research village, 8–10 community members, with a team of facilitating researchers and the aid of satellite imagery, created a participatory map of past and present land cover and land use. While the FGD was open for all to attend, primary participants comprised both male and female adults and were selected by community members at an opening community meeting for their knowledge of area land uses, land cover changes, and land tenure arrangements.

### Key informant interview with participatory maps

We conducted 4–6 key informant interviews per village for a total of 30 interviews across research sites. Informants were selected by community members in the opening community meeting based on relevant knowledge and included knowledgeable community members, customary leaders, and village heads. Some key informants also participated in the participatory mapping FGD, but identical participant sets were not required.

During the interview we used the participatory map produced previously as a visual aid. Key informants were asked a series of questions for each land cover category identified in the mapping FGD, including who uses and manages the area, for what purpose, and according to what arrangement. Terms for access, use, management, and exclusion rights were adapted from Larson (2012) [50].

Responses were then coded by land user, which included individuals (often households), groups (delineated according to extended family, neighborhood, or site-specific sub-village groups), the entire village, or a user group extending beyond the village scale. Combining remote sensing and participatory mapping data, spatial areas were coded based on this typology of user group.

While the larger data set includes valuable nuances regarding diversities of land uses, resources, temporalities, and user groups, for this paper we focus on spatially distinct areas, identified by key informants, that more specifically hold individual claims (for example a personal agricultural field), group uses (for example a hereditary fruit tree grove), village-level claims, or otherwise. Key informants were also asked for their perceptions of villages’ ability to access land and resources in the past, present, and future, with responses coded according to whether access for each land category was considered relatively difficult, easy, or conditional. These responses are summarized and presented in site-specific Results sections entitled “Perceptions of Land and Resource Access.”

### Household survey

We conducted 62 household surveys in Papua, 171 in Central Java, and 185 in West Kalimantan. The sample size in each village was defined by a function of population size, a confidence level of 95%, a margin error of 0.1, and a variance of estimated change. We used a simple random sample stratified by sub-villages to determine the sample size and randomly selected via lottery method. In the household survey, we asked multiple questions to assess household human capital; socio-economic conditions and trends; land uses; proximity to forest and mobility; and participation and motivation involving in local organizations. One section of direct relevance for our tenure analysis concerned respondents’ land uses, household rights over those land uses (whether ownership, ability to rent, or simply use), land right claiming processes, and particular land right agreements.

Combining these methods with an analysis of Indonesian statutory forest policy and Ministry of Forestry land classifications [49], we present three distinct lived land tenure contexts in the Results section.

## Results

With attention to both similarities within research sites and differences across site villages, the results below illustrate three distinct lived tenure contexts across our research sites in Central Java, West Kalimantan, and Papua provinces. The tenure contexts include: *negotiated partnerships* in Central Java between local communities and private timber companies; *tenure mosaics* in West Kalimantan comprised of simultaneous, overlapping statutory and customary claims to land and resources; and *customary authorities* in Papua comprised of overlapping and flexible claims based on the settlement history of the area.

**Fig 2:**
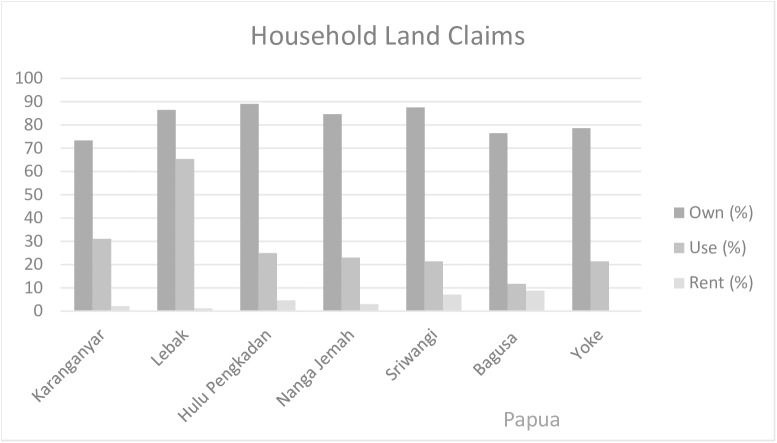
Absolute and relative frequency of households who own, rent and/or use piece(s) of land by villages in 2013–2014 (total n = 418).

Interesting similarities and differences in household perceptions of land rights are seen across research sites, captured in the HHS using categories of ownership, rental, and use rights (depicted in Fig 2). In all villages, a majority of households perceived that they owned the land they used for growing crops, fruit trees, or timber (more than 73–90% households in all villages across three sites), and very few households perceived that they rented land from someone else (0–9% of households). Households did differ, however, in the proportion who perceived themselves to only possess use right. Proportions range from 11% households in Papua to 67% in Central Java. This figure is useful to understand how many people in the village partake in a specific land or resource claim. Yet more so, in the range of respondents answers we see a difference in the understanding of ownership and use rights, requiring a more nuanced understanding of practiced tenure as described below. Significantly, there are lands respondents may utilize for various purposes, including hunting and collecting timber or non-timber forest products which they did not categorize as owning, using, or renting. Such differences both between perceived rights and across research sites suggest valuable differences in land tenure contexts. Combining the household surveys with the participatory mapping FGD and key informant interviews uncovered prominent site, scalar, and stakeholder differences across contexts and raised substantial questions for integrating PMRV within them.

### Negotiated partnerships in Central Java

According to key informants, land and resource rights in the villages of Karanganyar and Lebak are largely organized as individual land claims across agricultural, agroforestry, and timber lands. They understand these claims as ownership, representing a full bundle of rights: to access, use, and manage the land; exclude others from it; and sell the land to other people. Individuals buy land from other people or inherit it from their parents, representing this ownership with a land certificate issued by the government. Timber stands of predominately pine, mahogany, and teak, documented in participatory maps, account for just over a quarter of the total village land. These stands, legally classified as Production Forest within the Forest Estate, are currently managed by a state-owned timber company, Perum Perhutani, with whom villagers have a Forest Partnership agreement (Pengelolaan Hutan Bersama Masyarakat (PHBM)).

As described by key informants and FGD participants, PHBM is a negotiated partnership resulting from a history of conflict and compromise involving area communities, Perhutani, local NGOs, and the sub-district government. Following Indonesia’s national decentralization movement, in the late 1990s 30 villages in Wonosobo, including Lebak and Karanganyar, negotiated for rights to Perhutani forest. Leading up to this agreement, members of surrounding communities had logged much of Perhutani forest without the company’s authorization. Through the establishment of PHBM in 2001, Perhutani land was divided and distributed to those communities Perhutani categorized as a forest village community, meaning villages bordering their plantation stands whose livelihoods are to some extent related to forests. All households of Lebak and a portion of Karanganyar, whose area borders the plantation, were granted individually-based use rights to Perhutani forest land (See Fig 2). Key informants describe these rights to include (1) harvesting and selling certain timber species, sharing the profits with Perhutani (villagers retain 25% of profits from most timber species), (2) tapping pine trees for resin to be sold to Perhutani, and, (3) intercropping annual crops between timber trees. In return for these rights, villagers must help maintain their land parcel and monitor that trees are only cut with company approval.

Participatory maps show that agroforest and agricultural lands, held as individual properties, account for 66% and 68% of land in Karanganyar and Lebak, respectively. These lands are legally classified as Non-Forest Estate and individuals hold legal land titles issued by the National Land Agency (*Badan Pertanahan Nasional*).

Household survey responses reflect that households cut timber in both private parcels within Perhutani’s timber plantations and individually-owned and managed agroforests. Key informants describe that, within both land areas, tree species are managed for timber production, with the exception of fruit trees. Additionally, households collect NTFPs from both land types, however the varying lands yield different resources. At the time of our survey, between 9% and 14% of households in Karanganyar and Lebak, respectively, use Perhutani plantation forest land for either subsistence or cash income (Fig 1). Key informants express that the relatively low use of these areas reflects a temporal variability in timber plantation use, in this case, due to tree stands being too dense to cultivate crops in the understory but not yet mature enough to harvest. Comparatively, a greater number of households derive income from croplands, primarily paddy, and agroforests planted with fruit and timber trees. In Lebak, 72% of households receive subsistence and cash income from agroforests and 74% from crops. Figures in Karanganyar are lower, but still substantial, with 57% of households utilizing agroforests and 45% croplands.

#### Perceptions of land and resource access

In both Lebak and Karanganyar, key informants’ perceptions of access to land and resource rights follow two simultaneous narratives: that of increased land rights through the Forest Partnership (PHBM) agreement with Perhutani and decreasing agricultural land availability due to a fixed amount of land for an increasing population. Key informants reflected that prior to the establishment of PHBM in 2012, villagers were restricted from accessing Perhutani land or collecting any forest products. The area was tightly patrolled and if caught all resources were confiscated. Informants find future access conditional on Perhutani’s willingness to continue the Partnership agreement. Furthermore, villagers are unclear as to the time frame of the PHBM contract: time frames mentioned range from between 30–50 years to indefinite. However, the villagers feel they have a certain amount of leverage to maintain access, recognizing that Perhutani needs their help to maintain the village stands and protect them from unwanted logging.

### Tenure mosaics in West Kalimantan

Land and resources in the villages of Hulu Pengkadan, Sri Wangi, and Nanga Jemah in West Kalimantan interact across a weaving of individual, family, group, and village claims. The means to make and defend these claims rely on sources of legitimacy granted at the village, sub-district, or national level, creating a mosaic of overlapping and simultaneous land and resource claims.

Key informants describe forest land never cleared for agriculture, locally known as *rimba*, as managed and regulated by the village government. They likewise explain that customary rules grant villagers the right to harvest forest resources according to their needs, a right that can be granted to outsiders with permission from the village government. Household surveys show that within the *rimba v*illagers collect NTFPs, including rattan for weaving and wild vegetables, as well as timber for personal use.

Participatory maps show *rimba* accounting for 90% of land in Nanga Jemah, while only 12% in both Hulu Pengkadan and Sri Wangi. Such disparities might account for why some households in Nanga Jemah, due to a substantially larger forest area, also cut timber for commercial purposes. Without a business license required to sell timber and non-timber forest products, however, all products sold are limited to the informal market. All told, household surveys show that 77% of households in Nanga Jemah (more than any other village in our study) collect forest resources to sell.

FGD participants and key informants explain that many land and resource rights derive from the practice of rotating agriculture, in which the act of clearing forest establishes a largely private land claim. We categorize this as ownership in Fig 2. As the land rotates from upland rice to fallow and then onto rubber garden or secondary forest, the land remains under the land claim of the individual who opened it. Meanwhile, claims to planted tree groves or individual trees, primarily durian (*Durio sp*.), tengkawang (*Shorea sp*.), or tapang honey trees (*Koompassia excels*), belong to the person who planted the tree or their descendants. Depending on when the tree was planted, the descendants may be recognized today to include a single family, a neighborhood, the entire village, or outside communities with hereditary ties. These tree resource claims overlap and coexist with the private land claims made in clearing land.

Participatory maps display that individually claimed land accounts for 84%, 38%, and 10% of the total land area in Hulu Pengkadan, Sri Wangi, and Nanga Jemah, respectively. The majority of households (66%) in Hulu Pengkadan rely on cash income and subsistence from agricultural lands, while far fewer (31% and 37%) do so in Sri Wangi and Nanga Jemah, respectively. This is in contrast to the high degree of subsistence and cash income earned from agroforests, primarily rubber and tengkawang, by 94%, 93%, and 78% of households in Hulu Pengkadan, Sri Wangi, and Nanga Jemah, respectively.

Key informants describe that within all villages most claims are known by word of mouth; however, some individuals do hold written land deeds, issued and recognized by the village government to be used in land dispute cases. The village land boundaries are currently under negotiation between village leaders, which will be written down and approved by the sub-district government.

#### Perceptions of land and resource access

In West Kalimantan, key informants’ perceptions of access to land rights from the past to the present focus on the villagers’ ability to access enough agroforestry and agriculture land to support their livelihoods. The informants agreed that in the past accessing lands for agricultural and agroforestry production was not difficult; there were large forested areas to be converted, low land prices, and less people seeking land. Pre-established land rights, from opening their own land or inheriting it, are considered rights in perpetuity or *hak mutlak* (absolute rights) and are defendable in customary court. Yet when people move to the village or desire additional gardens, it is difficult today to find land. Villagers are not allowed to open new forest land in Hulu Pengkadan or Sri Wangi due to rules established and enforced by the village government. These limitations do not exist in Nanga Jemah where villagers are still allowed to open new land; however, informants noted that finding an area that is not already claimed is challenging and will be far from the village settlement.

The ability to access forest resources in the future is perceived to be uncertain. The respondents agreed that villagers will respect and follow the village’s management rules, including not being allowed to open new land in two of the villages. Yet some respondents question if the rules will be enough to sustain resources as villagers are still allowed to harvest timber and non-timber resources as they need. Despite local respect for village rules, other forest user groups threaten future land and resource access. The primary threats our informants mentioned included restrictions on community land use enforced by the national government, based on statutory forest classifications, and/or from companies with a forest concession.

Notably, over the span of this research, Sri Wangi and Nanga Jemah were granted approval for a Village Forest (*Hutan Desa*), which recognizes certain statutory legal rights to the forest. The official permit from the district remains incomplete, however, and it is not yet clear to key informants what legal rights will be recognized through the designation. Recent negotiations to establish the Village Forest boundaries in Sri Wangi led to confusion over statutory forest classifications in their village areas. This raised concerns over the potential restrictions on community land use, such as prohibiting forest resource extraction due to a lack of community statutory rights. Our respondents ultimately hope that the statutory rights gained in establishing the Village Forest will allow them to maintain their management systems and land access into the future.

### Customary authorities in Papua

Key informants describe land tenure claims within Bagusa and Yoke as governed through customary (*adat*) systems, organized along family lineages and shaped by ongoing social relationships. The Mamberamo Raya Regency where Bagusa and Yoke are located encompasses 59 villages found along the Mamberamo River. Yoke is comprised of two settlements, one along the coast and the other in the mangrove forest. Bagusa is a single settlement located in the swamp forest further upstream along the Mamberamo river. Villages are connected by ethnic lineages (*suku*), creating an interconnected *adat* system in which village boundaries are not strictly separated. Village territories are defined as the furthest area in which villagers have the right to hunt, fish, and collect resources. However, such tenure rights, whether it is to use rivers, lakes, or forests, often overlap between villages and across village boundaries.

Village-level claims to access, use, and manage land follow a historically-based hierarchy of settlement, with Yoke and Bagusa recognized as the first settlers and thus the oldest villages in Mamberamo Hilir District. Tenure disputes, both within and between villages, are mediated through customary leaders (*Ondoafi*), whose authority is hierarchically nested, following this same settlement history. Individuals and groups outside this shared ethnic lineage, unless joined by marriage, must first ask the appropriate *Ondoafi* for permission to collect resources or cultivate land within any particular village territory or territories.

Within villages, the territory is often divided and controlled according to the major *suku* groups, each with their own distinct language and culture. *Suku* ethnicities are further sub-divided into clans or *marga*, which are organized by family name [51]. Sacred spaces, gardens, and sago groves are often devolved to and managed at the *marga* level. Enforcement of such *suku* and *marga* based claims can be flexible. For example, while Yoke’s founding story begins with the marriage of a man and woman from separate Paito and Bosumbaso *suku*, the village has dissolved these ethnic bonds and the territory divisions that customarily come with them. This flexibility of enforcement is also seen in *marga*-based claims: there are cases of claims being strategically defended during times of conflict, while the same claims may dissolve during times of hardship such as a natural disaster.

Individual rights are patrilineal and passed down through *marga*, dictating where individuals can open their gardens, as well as which sago groves, forest areas, and water bodies they are allowed to collect from and are responsible for protecting. These are categorized as owning land in Fig 2.

Villagers’ gardens, cultivated as agroforests, include a variety of species, from banana, coconut, and areca nut palm (*Areca catechu*) to annual crops such as cassava. Sago (*Metroxylon sagu*), the regional staple food, is gathered from separate sago groves, which may be both wild and domesticated. At the time of our surveys, 65% of respondents in Bagusa and 75% in Yoke use gardens or groves for personal or commercial purposes. Households derive cash-based income from selling forest products and fish, as well as subsistence from myriad forest activities, whether hunting pig, harvesting eggs, or collecting sago. Only 7% of households in Yoke and 21% in Bagusa do not draw either cash income or subsistence needs from the forest. This figure excludes income and subsistence needs from collecting fish, which may come from both the sea and fresh water areas located within the forest. Timber extraction, as described by informants and confirmed in surveys, is minimal in both villages, with timber harvested as needed for housing, boating, and construction, and neither selling it commercially.

#### Perceptions of land and resource access

In Bagusa and Yoke, perceptions regarding the ability to access land and resources display a strong sense of continuity across time, with each key informant expressing confidence that customary laws are the most effective means to maintain village land rights, in the past, present, and into the future. Our informants described land and resource access as having been largely easy and conflict-free. However, respondents also noted that extractive companies have disrupted this lack of conflict surrounding land and resource access over the years. One example of this came from Yoke informants who spoke of a negative experience with the Mamberamo Shell oil company in the 1980s. Villagers describe how the company dug large channels through the community’s mangrove forests, causing sedimentation and killing many fish, all while providing the village with little compensation or restitution. Such experiences make villagers especially weary of the risks companies pose to future resource access and ecosystem health.

In thinking about the next generation, respondents reiterated the importance of maintaining the customary system for future land and resource access. In particular, they reflected on the need to maintain customary methods of conflict resolution, arguing that government and police involvement should be reserved as a last resort.

## Discussion

The local framings of negotiated partnerships in Central Java, tenure mosaics in West Kalimantan, and customary authorities in Papua, understood within the pluralist governance history presented in Section Two, show substantial variation between and within tenure contexts, as well as in perceptions of tenure security. Means of claiming across contexts are seen to vary dramatically, from clearing forest land, planting a resource, or marking a tree to local land letters, statutory land titles, proof of origin certificates for trees, or oral or written use agreements. The mechanisms for defending these rights also vary substantially across local, sub-national, and national scales–from customary negotiations in Papua to customary court or village proceedings in West Kalimantan to formal land titles or formally negotiated partnerships in Central Java.

We argue that a detailed understanding of lived land tenure contexts, such as those presented in the results above, is required not simply for reasons of equity, but also practicality, as attempts to negotiate claims within such systems will have ramifications for both local communities and the effective pursuit of REDD+. Boyd ([52] p. 911) argues that the success of REDD+ hinges on the ability to insert such abstracted, transnational toolkits as MRV into situated socio-ecological contexts, to establish appropriate and “specific legal and institutional forms in particular places.” The legitimacy of such toolkits, scholars argue, rests not only on local participation within MRV activities, but crucially also on the consideration of local knowledge and rights [3,53]. Attention to lived tenure contexts will not only engage systems of tenure as they are practiced, but it will also enable an MRV system that is “(responsive) to local needs and diversity, where the local is actively re-enrolled in knowledge infrastructures and practices of environmental governance” ([3] p.729).

A primary benefit of local participation in MRV discussed in the literature is its capacity to adapt MRV standards and methods to particular social and environmental conditions due to the inclusion of local communities and their situated and specialized knowledges [54]. With this in mind, we argue that attention to local land tenure contexts is essential for the construction of an accountable and legitimate Participatory MRV system. In considering how local land tenure contexts potentially influence MRV standards and methods, we raise three relevant and arguably fundamental concerns: (1) who has authority to conduct PMRV activities, (2) how are communities differentially impacted by PMRV, and (3) what influences motivation to participate in PMRV.

### Who has authority to conduct PMRV activities?

The success of PMRV in some sense rests on answering a deceptively simple question, namely *who* can measure forest carbon *where* across a particular forest landscape. Studies on participatory monitoring provide a series of considerations for how to select who should measure forest carbon. Community-driven participant selection is thought to enable measurement and monitoring activities that operate within, and respect, existing sociocultural relationships [55]. Such activities, however, can also risk reinforcing existing power relationships [55], pushing some researchers to recommend the intentional selection of locally marginalized groups such as women, the impoverished, elders, or the illiterate in order to broaden participant representation and attend to issues of equity [56].

Lived land tenure contexts present additional complexities regarding who can measure forest carbon and where. As presently envisioned, MRV involves the establishment of long-term monitoring plots to effectively measure forest carbon and its fluctuations over time. Our results raise considerations for the identification of who has the right to measure within those plots, as well as who has the authority to grant permission for that measurement. Indeed the desired plots may include not only multiple actor groups holding a variety of statutory and customary resource claims but additional nuances of how and when such claims are enforced.

Our sites in West Kalimantan display the risk of complications due to an absent legal right holder, according to national concessions. The logging company is still considered the legal right holder of the majority of forest land despite being inactive on the ground for more than 10 years. When working through a national legal framework the existing logging concession’s rights prevents the possibility for local people to participate in and benefit from REDD+ and MRV in this forest.

Yet another challenge to this question of who measures where concerns the heterogeneity of local communities within particular lived land tenure contexts. Previous scholars’ work interrogates the concept of a community to extend beyond a small spatial area, a homogeneous social group, and a set of culturally shared norms [57]. Instead, these authors note, and our results support, that communities can possess expansive and porous spatial boundaries; heterogeneous social, economic, and political structures; and actors with diverse needs, priorities, and identities. The village sites in Central Java and Papua illustrate two strikingly different examples of how such concerns can manifest across particular land tenure systems and communities–and what that might mean for PMRV.

In the negotiated partnerships in Central Java, the formal and individualized nature of claims will shape who has authority to measure particular designated MRV plots. Interestingly, while such a system could bring welcome clarity to questions of participant selection and authority, this same system could pose a potential obstacle for reaching a consensus to participate in MRV across private landholders. Additionally, measurements in land under a Forest Partnership agreement will require renewed negotiations of rights and benefits across Perhutani and village stakeholders and raises substantive concerns regarding who can claim the right and authority to both measure and choose where to measure on Partnership land.

The local tenure system seen in the Papua sites offers its own set of complications regarding PMRV participant selection and authority. The customary system is one that is fundamentally interconnected and flexibly grants overlapping resource access, use, and management rights across multiple communities. The lived land tenure context as described by key informants can thus be viewed as not simply about relationships between people and resources but rather as complex relationships between people themselves [26,30]. The lack of clearly delineated spatial and social boundaries poses distinct challenges for PMRV, as it presents a concept of community that extends beyond a single village, as well as the reality of porous and overlapping village territories. Siting monitoring plots within territories with overlapping village rights and responsibilities can create logistical dilemmas regarding how measurement activities are distributed within and between villages. One potential outcome of such uncertainty is double forest carbon accounting. Another potential outcome of implementing PMRV in these overlap zones is that certain actors could try to solidify particular claims or exclude other users through formal or informal means.

Whether the social, economic, and political differences across Forest Partnership stakeholders in Central Java or the filially-connected and flexible nature of customary tenure in Papua, we see contexts that confound traditional understandings of community and highlight heterogeneities that will need to be negotiated for the design and enactment of equitable and effective PMRV.

### How are communities differentially impacted by PMRV?

The uncertainty of how REDD+ carbon rights will be defined in Indonesia complicates efforts to design PMRV-related compensation and benefit-sharing mechanisms. It is as yet unclear how assigning carbon rights in REDD+ relates to pre-existing systems of land or resource rights [58]. Carbon rights may be recognized as linked to a particular resource, providing those with rights to that resource with rights to its carbon as well. This distinction opens notable areas for conflict when considering differences between above and below carbon sources. Alternately, carbon rights may be fully designated to nation-states. This would open up the possibility for carbon rights to be separated from rights to other resources, creating space for substantial conflict around such claims and the associated benefits [59,60].

Right now participation in MRV does not equate to benefiting from carbon rights; however, Larrazábal, et al. [19] argue that one key potential benefit of PMRV is as a source of local empowerment by giving communities ownership of carbon data and thereby increasing their negotiating power for carbon rights. Other scholars have argued that the statutory recognition of local communities’ carbon rights in REDD+ can serve to legitimize the communities’ greater tenure rights and claims, helping to secure tenure for the long-term [28].

Navigating carbon rights is only more complicated when placed in conversation with lived land tenure contexts. In Central Java’s Forest Partnership there is a need to clarify how local communities’ use rights to Perhutani land relate (or not) to carbon rights and related benefits. The mosaic of tenure rights in West Kalimantan raises the important question of which legal scale–national, sub-national, or village–will hold the authority to determine the connection between tenure claims and carbon rights. Key informants in Papua, meanwhile, suggest that carbon benefit distribution would be most clear and culturally attentive if it operated according to the existing customary village hierarchy. The overlapping tenure system across village territories there, however, potentially opens up substantial space for confusion in tracking causation of forest carbon fluctuations across forest user groups.

Our results reveal the potential for high variation both between and within villages regarding the amount and type of land claimed, as well as the way that land is used. Our West Kalimantan sites offer a powerful example of how such variation may complicate PMRV activities and have differential community impacts. This is particularly seen in the potential of monitoring forest carbon and forest carbon change to highlight certain commercial forest uses considered illegal under statutory law. While such activities might complicate or compromise the accuracy of relevant forest carbon measurement, alternatively, efforts to mitigate these activities will have varied effects across individuals in the village, as people are seen to be dependent on forests to varying extents and for varying needs. Such costs show that any PMRV system will have varied economic, social, and political impacts within a community. These variations should be considered when designing and implementing PMRV in order to identify culturally appropriate incentives and benefit-sharing distributions for PMRV. This foresight may help to recognize potential arenas of conflict and negative impact for those village groups and individuals without decision-making power.

### What influences motivation to participate in PMRV?

Securing tenure is seen to act as a significant incentive for local participation in community conservation efforts and potentially extends to REDD+ and PMRV engagements [58]. Tenure security is defined as the certainty that a person’s rights to land and resources, across both de jure and de facto realms, will be recognized and protected in the present or future [12]. Social developmental MRV systems have been hailed as opportunities to secure community land and resource rights, and help ensure procedural rights such as free, prior, and informed consent (FPIC) [61]. The relationship between tenure security and PMRV participation, however, is complicated by the potential for such security to empower communities to participate in GHG-emitting rather than GHG–mitigating economic activities [12]. The diversity seen across the lived land tenure contexts above holds important lessons for unpacking the relationship between tenure security and motivation to participate in PMRV.

Local perceptions of access to land and resources serve as an indicator for villages’ desire to increase tenure security and, if so, through what means. Despite recognition that there is not necessarily a link between tenure security and statutory land titles [62], discussions around land tenure and forest carbon are often framed in terms of the presence and absence of community statutory rights [6,63]. Our results show that the shifting roles of statutory and customary authority in Indonesia, as displayed in Section Two, unsettle this polarity.

In examining tenure security across our three research sites, we detected noticeable differences in the relationship between perceived security and statutory rights. Specifically, when looking at local perceptions of future land access, we found that tenure security does not always correlate with the extent to which practiced tenure arrangements are recognized in statutory law. Rather we find that perceptions of tenure security are dependent upon by whom actors feel threatened and in need of defending their tenure claims against.

We see across all three sites the presence of lived tenure contexts that have clear and robust methods for claiming and defending rights within and between neighboring villages. Yet, importantly for the consideration of PMRV, the basis of these claims varies across sites: in Java defending claims depends on statutory land titles; in Kalimantan on a combination of verbal and written claims upheld by the village or sub-district governments; and in Papua on understood ethnic and clan claim histories.

The negotiated partnerships in Central Java offer an example of where increased tenure security has already been used as a tool to motivate community members to participate in forest monitoring activities. The PHBM Forest Partnership provides forest-adjacent households limited use rights in exchange for managing the land and monitoring unapproved extraction. In this example, the incentive of tenure security was directly related to how increased forest and resource access enabled additional, non-monitoring related livelihood benefits. Despite legal recognition, Central Java key informants note that future access to land and resources is uncertain.

While considered defensible at the local level, land and resource claims in West Kalimantan and Papua may be considered insecure when confronted by obstacles beyond this level. In West Kalimantan we see that tenure is considered most insecure when confronted by claims from the central government, which does not legally recognize their land and resource claims outside the newly approved Village Forest (*Hutan Desa*). According to national law, the legal Forest designations shape which land uses will gain this statutory recognition. A Village Forest can only be located in the absence of exiting statutory resource or land concessions. With most of the Production Forest land within the villages still under the forest concession from the 1980s (See Table 1), the Village Forest is primarily located in Protection Forest.

In Papua, key informants did not feel threatened by the legal forest classifications and resulting land use restrictions from the national government. Key informants described the practiced land tenure and land uses in terms of customary authority and did not reference official land classifications or statutory claim recognition. According to the national government classifications, the majority of both village areas are designated as Forest Estate, with only small areas carved out as settlements and designated as Non-Forest Estate. Yoke’s territory, beyond its settlement area, is part of a wildlife reserve. This classification as a Conservation Forest statutorily prohibits hunting, fishing, gathering, and timber extraction. Bagusa’s territory, on the other hand, is largely under Production Forest with an active logging concession providing the company PT Mamberamo Alas Mandiri rights to extract and sell timber.

Whether buffered by historical ties to the land, or Papua’s status of Special Autonomy, community members expressed confidence in their customary claims to land and resource access and the maintenance of those claims. Where they did express concerns over tenure insecurity, however, was in speaking of the threats posed by companies engaged in resource extraction. Local communities spoke of personal and greater area experience with certain commercial actors; the distrust they felt towards them; and concerns over potential illegal land and resource encroachment in the future.

Looking across our cases, we see communities perceive tenure threats from both government and corporate actor groups, whether they statutorily possess secure tenure, as in the Java example, customarily as in Papua, or in more hybrid arrangements such as West Kalimantan. These examples show how such concerns and distrust can influence perceptions of tenure security.

Whether with corporate or governmental actors, NGO practitioners or academic researchers, such perceptions of trust are seen to be important in advancing sustainable relations with local and indigenous communities [64,65]. Recent ethnographies highlight how feelings of mistrust and betrayal can arise from misunderstood expectations between community and conservation actors, stymying and endangering the fates of conservation and development projects [66–70]. The effects of such tensions point to the importance of understanding histories and inequities between communities and outsiders. They also highlight the need for programs such as REDD+ and activities like PMRV to interrogate models of participation that can otherwise ignore such histories, reify existing power structures, and contribute to conflict between communities and conservation actors [70–72]. The cases above show how legitimacy arises from more than formal legal agreements. Motivation to participate in a *legitimate* PMRV will be affected by consequential, informal relations of trust as well.

### Minimizing conflict

The discussion above highlights three ways PMRV must grapple with lived land tenure complexities: negotiating questions around PMRV rights and authority; potential differential impacts of PMRV within and across communities; and locally situated linkages between tenure security and the motivation to participate in MRV. Though we argue these considerations are necessary to shape a legitimate PMRV system, they are not sufficient. Given the fact that quantifying forest carbon will necessarily advantage certain groups of people over others, there is still substantial potential for conflict to arise in PMRV activities. This gained advantage could occur in many forms–from secured land tenure to recognized carbon rights to social and political empowerment, all at the potential expense and exclusion of others.

Local conflict resolution mechanisms are critical then to arrive at solutions deemed appropriate and legitimate by local people. It is for this reason that we argue for increased and nuanced attention to local systems of tenure arbitration and their adaptation and inclusion in PMRV design and implementation. It is important to recognize, however, that the quantification and commodification of forest carbon through REDD+ has the potential to create new spaces of conflict, whether regarding the distribution of benefits, the ownership of carbon, or novel means for making and defending tenure claims, and that such conflicts might not be resolvable through local means. This too is a reason to examine more carefully and grapple more attentively with the pluralistic reality of lived land tenure contexts in countries like Indonesia.

## Conclusion

This paper investigates how consideration of local *lived* land tenure contexts can contribute to crafting”legitimate” [3] and participatory MRV systems. Specific land tenure contexts in Indonesia’s Central Java, West Kalimantan, and Papua provinces show the importance of dealing with the reality of legal pluralism across REDD+ landscapes. In particular, the examples of negotiated partnerships, tenure mosaics, and customary authorities allow us to view both a diversity of livelihoods and a range of strategies employed by local people to make and defend land and resource claims across research sites. Taken together, such tenure contexts also let us recognize the heterogeneity of communities that will be engaged and implicated in PMRV, as well as the lack of clear boundaries between customary and statutory systems and feelings of tenure security and insecurity related to them. Additionally, the tenure pluralism presented here highlights several critical areas of consideration for future effective and equitable PMRV design and implementation.

Specifically questions of who holds PMRV rights and the authority to grant them; how PMRV might differentially impact individuals in a community; and what the relationship is between tenure security and motivation to participate in MRV activities are critical to consider when designing PMRV to fit local land tenure contexts. Village forests are seen to contain various sources of authority, whether through statutory or customary claims, that could be capable of permitting PMRV activities. These claims speak to historical and contemporary social relationships and levels of community that must be considered when participants are selected for MRV activities.

Additionally, benefit division and distribution must consider the heterogeneous nature of communities affected by PMRV, including the variation between the amount and type of land and resource claims made by different user groups and the forest-based livelihoods found within a village in order to avoid creating conflict and negatively impacting individuals who may not be in decision-making positions.

Finally, increased land tenure security may act as an incentive for participation in MRV activities and REDD+ goals; however we see that such an incentive is contextually dependent. Two specific examples that arose in our research include whether local communities perceive their tenure as insecure, as well as whether secure tenure rights lead to improved local livelihoods. Importantly, perceptions of tenure security and insecurity do not always correlate with statutory rights recognition, and informal relations of trust can influence perceptions of governance legitimacy in potentially relevant ways.

Taken together, we see that considering PMRV in distinct lived land tenure contexts presents multiple potential sources of conflict, whether between individuals in a village, neighboring villages and company partners, or various legal and customary sources of authority. Given this, we argue local conflict resolution mechanisms are key to arriving at solutions deemed appropriate and legitimate by local people.

One remaining question regards implementation lessons for REDD+ practitioners. While this paper derives from a research project focused on PMRV enabling conditions and feasibility, rather than project operationalization, we nevertheless offer reflections on how implementers can best integrate the concept of lived land tenure contexts into PMRV engagements.

Our paper highlights the importance of utilizing mixed methods to uncover the diverse ecosystems, land uses, and tenure regimes operating in lived land tenure contexts. Bringing methods from the social, geospatial, and ecological sciences together will allow for multiple scales of data to be collected. It also enables the pursuit of an expansive evidence set from the natural and social sciences and across quantitative and qualitative metrics.

The diversity, complexity, and dynamism of lived land tenure contexts, however, reveal challenges for any attempts to clearly delineate, simplify, or standardize methodologies for uncovering them. More fundamentally, the place-based nature of land tenure contexts reinforces scholars’ calls for contextuality in understanding REDD+ landscapes and corresponding interventions. While such realities might be considered barriers to designing scalable yet tenure-appropriate PMRV systems, we instead argue that they offer an opportunity for advancing the role of local and indigenous collaboration in understanding and adapting lived tenure contexts to MRV activities. By working with local and indigenous partners, valuing their knowledge systems, and cultivating relationships grounded in humility and respect, collaborative processes can help uncover the multi-scalar nature of claims, as well as how indigenous ecologies and worldviews shape tenure regimes.

In considering all of this, it is important to note, however, that the quantification of forest carbon presents a potentially new commodity and emerging market, as well as a new object for claiming. In response, new claiming methods may emerge that might not accord with current local conflict resolution mechanisms. Further research into such mechanisms and how to integrate forest carbon claims within them is vital. Ultimately, this paper is merely the beginning of attempts to unpack the impact PMRV may have on local communities, their livelihoods, land management practices, and land tenure arrangements. Further attention to and unraveling of these topics is essential for the effective and equitable design and implementation of PMRV both in Indonesia and around the world.

## Supporting information

S1 Fig Household Survey Page 1–7. (PDF)

S2 Fig Household Survey Page 8–9. (PDF)

S3 Fig Key Informant Interview Land Tenure Questionnaire. (PDF)

S4 Fig Participatory Mapping Questionnaire. (PDF)

S5 Fig Participatory Mapping Questionnaire Table. (PDF)
