# Supplementary material for: Considering land tenure in REDD+ participatory measurement, reporting, and verification: A case study from Indonesia
Source: PLoS One. 2017 Apr 13;12(4):e0167943. doi: 10.1371/journal.pone.0167943 (PMC5390967; doi:10.1371/journal.pone.0167943)
Supplement: S1 Fig — (PDF) [file pone.0167943.s001.pdf]

Desa : Nama Responden :
Tanggal : Page : /

Willingness

No	Question	Yes/ No	Explanation
30	Do you participate in one or several groups or organisations in this village? (e.g. farmer group, cooperative, posyandu, PKK, etc)<br><br>*Further question:*<br>- What is your motivation to **participate** in one or several groups or organizations in this village?<br>- What is your reason reason **not to participate** in one or several groups or organizations in this village?	Yes/ No	*(if respondent participate in several groups and organizations, ask his/ her motivation for involving each oranization)*
31	What are the names of the communities or organisations?		
32	Are you theofficial of communities or organisations that you involve in?<br>(e.g. farmer group, cooperative, posyandu, PKK, etc)	Yes/ No	(If yes, what is your position?)
33	Do you participate in social activities in the village? (e.g.: cleaning the village, etc)<br><br>*Futher question:*<br>- What is your motivation to **participate** in social activities in the village?<br>- What is your reason **not to participate** in social activities in the village?	Yes/ No	*(if respondent participate in several social activities, ask his/ her motivation for involving in each social activity)*

Desa : Nama Responden :
Tanggal : Page : /

Trends in Natural Resources and Demographics

We want to know your opinion,
Do you agree, disagree, or don't know with these statements:

#	Statements	Agree	Disagree	Don't Know	Explanation
**Natural resources**					
34.	Soil fertility of cropland and forest are better now than 10 years ago.				
35.	Number of agroforestry/ *kebun*/ *dusun* are more than 10 years ago.<br>Agroforestry (Java), *kebun* (West Kalimantan) or *dusun* (Papua)				
36.	Fresh water is easier to get now than it was 10 years ago.				
37.	There are more hunted animals now than 10 years ago.				
38.	People are easier to get fish now than 10 years ago.				
39.	It is easier to find building materials from forest now than 10 years ago (timber, etc.).				
40.	People gather more NTFPs (edible plants, honey, resin, *kayu putih*, medicine, materials for handicrafts, rattan, etc.) now than 10 years ago.				
**Demographics**					
41.	The population of the village is larger today than 10 years ago				
42.	More people are working inside the village today than 10 years ago				
43.	More children in the village go to school than 10 years ago				

Desa : Nama Responden :

Tanggal : Page : /
