# Supplementary material for: Considering land tenure in REDD+ participatory measurement, reporting, and verification: A case study from Indonesia
Source: PLoS One. 2017 Apr 13;12(4):e0167943. doi: 10.1371/journal.pone.0167943 (PMC5390967; doi:10.1371/journal.pone.0167943)
Supplement: S2 Fig — (PDF) [file pone.0167943.s002.pdf]

Desa : Nama Responden :

Tanggal : Page : /

Household Survey Questionnaire						
Writer		Interviewer			Entered by	
					Checked by	
		Original or copy	O	C	File Name	
Checked by		Written in the back	Y	N	Been copied?	
Respondent		Village				
Gender		Hamlet		RT & RW		

Human Resources and Educational Knowledge

1. How old are you?

2. Where do you come from? (which village and city)

3. What is your marital status?

   a. Single

   b. Married

   c. Widowed

   d. Divorced

4. What is your latest education level you graduate?
   a. Never go to school
   b. Not graduated from Elementary School
   c. Elementary school
   d. Study Group for Package A
   e. Junior High School
   f. Study Group for Package B
   g. Senior High School
   h. Study Group for Package C
   i. University (Diploma/ Undergraduate/ Graduate)

5. Have you been in informal education (as training)?
   a. Yes
   b. No
   If yes, please mention one(s)

Desa : Nama Responden :
Tanggal : Page : /

6. **A.** Do you use cell phone daily?
   a. Yes
   b. No

   B. If no, have you used cell phone before?
   a. Yes
   b. No

7. Do you have any experience to use a computer or laptop ?
   a. Yes
   b. No

8. What is your main occupation? (only one, as: civil servant, farmer, cattle man, labour, trader, etc)

9. What is your any other occupation(s)? (can be more than one, as: "warung" owner, trader, etc)

10. (*If respondent work in forest and/ or cropland*) Do you? *
    a. Own it (own right/ hak milik)
    b. Rent it (rent right/ hak sewa)
    c. Only use it (use right/ hak guna)

    Explanation: (*) answers can be more than one

Notes: (*since when, until when, how big it is, how the owning/ renting/ using process, **if renting/ using:** to whom and what requirements you fulfil*)

Desa : Nama Responden :

Tanggal : Page : /

11. Information about **household members**

Name	Status within family (household head, husband, wife, son/ daughter, etc)	Gender M / F	Age	Latest graduated education level (received graduation certificate)	Occupation		Staying in the village (for how long a year)
					Main	Side	
a.						1.	Yes / No
						2.	
						3.	.... months
						4.	
						5.	
b.						1.	Yes / No
						2.	
						3.	.... months
						4.	
						5.	
c.						1.	Yes / No
						2.	
						3.	.... months
						4.	
						5.	
d.						1.	Yes / No
						2.	
						3.	.... months
						4.	
						5.	
e.						1.	Yes / No
						2.	
						3.	.... months
						4.	
						5.	
f.						1.	Yes / No
						2.	
						3.	.... months
						4.	
						5.	
g.						1.	Yes / No
						2.	
						3.	.... months
						4.	
						5.	
h.						1.	Yes / No
						2.	
						3.	.... months
						4.	
						5.	

Desa : Nama Responden :
Tanggal : Page : /

Name	Status within family	Gender M / F	Age	Latest graduated education level	Occupation		Staying in the village (for how long a year)
					Main	Side	
i.						1.	Yes / No
						2.	
						3.	.... months
						4.	
						5.	
j.						1.	Yes / No
						2.	
						3.	.... months
						4.	
						5.	
k.						1.	Yes / No
						2.	
						3.	.... months
						4.	
						5.	
l.						1.	Yes / No
						2.	
						3.	.... months
						4.	
						5.	
m.						1.	Yes / No
						2.	
						3.	.... months
						4.	
						5.	
n.						1.	Yes / No
						2.	
						3.	.... months
						4.	
						5.	
o.						1.	Yes / No
						2.	
						3.	.... months
						4.	
						5.	
p.						1.	Yes / No
						2.	
						3.	.... months
						4.	
						5.	

Desa : Nama Responden :
Tanggal : Page : /

Sources of Income

12. What are your household's sources of income during a year period? Please mention from the highest one (*including donation, government aid*)

a.	*Notes: (further description about sources of income; donation from whom, what is the name of the donation or government aid, etc)*
b.	*Notes:*
c.	*Notes:*
d.	*Notes:*
e.	*Notes:*

If your household gather forest, garden or crop land products (including plants and animals)

13. What are 15 main forest products that your household collect? (can be less than 15)	14. Is it for self-consumption? (non-*cash*) (Yes/ No)	15. Is it to be sold? (*cash*) (Yes/ No)
a.		
b.		
c.		
d.		
e.		
f.		
g.		
h.		
i.		
j.		
k.		
l.		
m.		
n.		
o.		

Desa : Nama Responden :
Tanggal : Page : /

Travel Distances and Modes of Transportation

No.		a. Forest*	b. Garden*	c. Cropland*
16.	Do you go to :	Yes / No	Yes / No	Yes / No
17.	What for :			
18.	How far is it for going to : **(unit of "km")**			
19.	How long does it take for going to: (**unit of "hour"**)			
20.	By what mean do you go to :			

Explanation (*):
Forest (*Hutan*) is an area with a batch of trees and other plants, both woody and non-woody, which occupies a certain area so as to create climatic condition in which is different to surrounding area
Garden (*Kebun*) is an area used by communities to grow various plants, primarily woody plants, for example: sengon, rubber, banana, sagoo, etc
Cropland (*Lahan pertanian*) is an area used by communities to grow various non-woody agricultural crops, for example: paddy, corn, etc.

No.		a. Market*	b. City*
21.	Do you go to :	Yes / No	Yes / No
22.	What is the name/ city of the market that you go to (*can be more than one*) :		
23.	Where is the market(s) located:		
24.	What for do you go to :		
25.	How often do you go to :		
26.	By what mean do you go to :		

Desa : Nama Responden :
Tanggal : Page : /

Explanation: (*) If respondent answers going to more than one market or city, ask one by one the following questions for each market or city

27. How long and/or far does it take to gather water from your home?
    (**metric units of "km" and/ or "hour"**)

28. Does the terrain or anything about your infrastructure make travelling difficult?
    a. Yes
    b. No

29. (Referring no.28) Why *(flat, mountainous, mangrove, riverine*)?
