# Supplementary material for: Considering land tenure in REDD+ participatory measurement, reporting, and verification: A case study from Indonesia
Source: PLoS One. 2017 Apr 13;12(4):e0167943. doi: 10.1371/journal.pone.0167943 (PMC5390967; doi:10.1371/journal.pone.0167943)
Supplement: S3 Fig — (PDF) [file pone.0167943.s003.pdf]

Village :   Name of Respondent:
Date :   Page :   /

Key Informant Interview: Land Tenure							
Writer		Interviewer				Inputed by	
						Checked by (in the database)	
		Original or copy	O	C		File Name	
Checked by (in the field)		Notes on the back of page (s)			Y	N	Copied?
Respondent		Village					
Gender		Hamlet				RT & RW	

Objective: The aim of this Key Informant Interview is to understand the land tenure arrangements as understood and practiced by villagers. It aims to identify stakeholders of different areas of land, inform power relations both within and outside the community, and assess perceptions of future land use and access.

Preparation:

Prepare Base Maps with GIS according to individual needs for each location. See guidelines.

Materials:

6+ Base Maps for Land Tenure (per village) *to be used for questions 1-4
1 Land Use Map at scale of above *to be used for question 6-7
1 Satellite Image at scale of above *to be used as reference
6+ Blank Land Tenure Tables
Pens for drawing new boundaries *check that pen color shows up on map

Key informants: (4-6) will be individuals from the village chosen for their knowledge on tenure systems including: traditional leaders, government representative, religious leaders, elders, village head, village secretary, individuals from the participatory mapping exercise, guide for ground check and more. The key informants should represent various relationships with the land and perspectives, including at least one person of each gender.

Village : Name of Respondent:
Date : Page : /

Duration: +/- 2 hours

Definitions:
Access rights: the right to enter the area.

Use Rights: the right to obtain resources, such as timber, firewood or other forest products, from the land.

Management Rights: the right to transform the resource.

Exclusion rights: the right to decide who can use the land and who is prevented from using the land.

Alienation rights: the right to sell, transfer or lease the land including the transfer of all rights to access, use, manage or exclude from the land.

(Above definitions based on Anne Larson (2012) Tenure rights and access to forests; a training manual for research. Part I. A guide to key issues.)

Land User: The individual or group with access and use rights.

Land Controller: The individual or group with exclusion rights.
*Either the land user or the land controller could have Management Rights over the land.

Arrangement: The arrangement of use or access (and possibly management) between the user and the controller.

Source of Authority: What source of power enables The Controller to regulate who has the right to exclude others from accessing, using or converting the land.

Village : Name of Respondent:
Date : Page : /

Guidelines:

To prepare for this key informant interview a Base Map should be created for each village in collaboration with the GIS team. This map will be display the land cover and/or land use information from the Participatory Mapping exercise. The information that is displayed on each map should fit the unique land use and cover conditions of each site, yet all maps will have the same format to allow comparative analysis.

*Due to the sensitivity of information, the interview should take place in a location where the informant will feel comfortable such as their home. Explain both our project and the topic of the interview. Explain to them that this map and this activity on land tenure is only a research activity, and will not be used for official documentation of land tenure. The map won't be used to develop RTRW or as proof in the settlement of any future conflicts. A**sk for FPIC.** Familiarize the informant with the Base Map by explaining that it is the result of a focus group discussion with members of the village and point out what the displayed colors represent and how we will use them (to identify various rules of land use and access).*

We are not asking the informant to verify information gathered in the participatory mapping exercise, yet any corrections they mention should be documented.

Directions on using the Base Map to fill out the Table:

The table should be carefully filled out to give spatial reference to the details given in questions 1-4. The interviewer should collect the information and create a system for indicated the area that is being referenced. This could be by numbering the polygons or drawing on the map with an index to collect what each new pattern indicates. New lines are drawn with a specific color to represent what type of information you are drawing (blue lines are different users, pink lines are different controllers, orange different arrangement, and green different authority). The legend should be drawn on both the map and the table. If multiple numbers have the same land tenure arrangement you can write "same as #_" in the field. If the key informant describes a new delineation and thus the polygon is split, the new sections become 1a,1b,1c etc.. Each new polygon should be given a new row or linked to a row with the same tenure arrangement.

Examples:
Polygon 1: A simple case will be if the Key Informant says a tenure arrangement corresponds to the Base Map polygon. All villagers are allowed to Use the land, and people from other villages are excluded from use. The adat leader regulates how the land is used (has management rights), and is the individual that regulates disputes over who can access the land so the Controller is the adat

leader. It is understood that all villagers are allowd to use the land, if there is a dispute among villagers it is brought to the adat leader. The adat leader claims Authority based on a customary claim that has existed in the village since it was founded.

Polygon 2: (Example of different users) This is a situation in which within one polygon the Key informant describes two sections: one where LMDH members are allowed to use it and the other in which all villagers use it under a customary claim. You draw where this division occurs and label the first section 2A and the second 2B. In the 2B row you can write "same as polygon 1" because User, Controller, Arrangement and Authority match the information filled in for Polygon 1. In the 2A row the key informant describes that some people from the village harvest timber even though they do not have an arrangement with perhutani. This information is written under "users that are unauthorized". Perhutani is the Controller because they decide who can use the land and how they are allowed to use it. They have management rights because they control what the LMDH members are allowed to plant on their land. The arrangement is that LMDH members must have a contract with perhutani and are required to provide part of their harvest to them. Perhutani has authority over the land based on a concession from the national government.

Polygon 3: (Example of different controllers). This is a situation in which the same group uses the area within a Base Map polygon there are a few groups which control access to this land. An oil palm company uses polygon three. Only employees of the oil palm company are able to use the land and it is only for company needs. Villager employees of the oil palm company only have access rights and do not have use rights as they are not able to use the harvests for their personal gain. The oil palm company has management and exclusion rights to this land through three groups: one is Pak X (area indicated as 3a) who the company rents from, part of the land is state forest land (3b) which has a rental agreement with the oil palm company and part of the land the oil palm company has their own forest concession (3c). Pak X claims this land based on a land title. The state claims this land as under the control of Ministry of Forestry. The Oil Palm company has a forest concession issued by the Provincial government.

Polygon 4: (Example of both different users and different controllers). The Key Informant describes that for part of the polygon (draw line and label 4a) all the villagers use it and for part the polygon (label 4b) it is the same arrangement as 2a in which LMDH has a contract with Perhutani. Under the row 4a write "same as 2a"). Within the polygon 4a part of the land is claimed as a customary forest and part of it is held in private land titles to villagers. Each of these sections is drawn on the map and filled with a symbol to represent the type of controller, star for adat and a triangle for private land. In the table 4a Star can be filled in "same as 1". For the 4a Triangle row, the controller is each villager over his own land which includes management rights, the arrangement is that each villager can only access his only private land and the authority comes from a land title.

Village : Name of Respondent:
Date : Page : /

Methods:
Through referencing the Land Tenure Base Map indicate a polygon and ask the following questions:
**Note: As the informant describes various tenure arrangements within one polygon, the interviewer should ask where on the map this occurs and in the appropriate color either draw a line and indicate 1a and 1a or give different patters to the two parts.*

1. Users:
   a. Who is allowed to use this land and how do they use it? (write the name of the group and what they do i.e. Villagers: access or LMDH: collect resin and plant certain agriculture species)
   b. Who is not allowed to use this land?
   c. Is there anyone who is not supposed to use the land but who uses it anyways and how do they use it? (e.g. other communities, companies, etc.)

2. Controller: Who grants access to or use of this land?
   Alternatives: Who is the land owner? Who is the land manager? Who controls resource use on this land?

3. Arrangement:
   a. How does the user get permission from the controller to access this land *(do you ask the adat leader, get a land title…)*
   b. What are the conditions of the arrangement? *(Does the user have use, access or modification rights? Does the user have to give anything to the controller in order to access/use the land?)*
   c. What is the length of this arrangement? (*Do you need permission every time you access, do you need to renew your contract every 20 years ect.,)*
   d. Can the users transfer these rights?

4. Source of Authority: What gives the person/group identified in question 2 the authority to control access and use? (contract with the government *(at which level, province/national)* , *historical legitimacy, victor of clan dispute ect.)*

Village : Name of Respondent:
Date : Page : /

5. Each site will investigate the reason certain land uses (see below) are located in a certain area. The information gathered should be general to the village rather than specific to the interviewee. *This will be dependent on the land uses indicated in the Participatory Mapping exercise*. *If the Land Tenure map does not display/list land uses, you should switch to the Land Use map to answer questions.*
   How is the land the village dedicates to ______ chosen?
      i. Agriculture: *Alternative questions: does the village choose where to put kebun by good soil, by the distance from where they live, because their family inherited that land or any other reason?* Or *why is agriculture located here and not in this other location which seems closer to the village?*
   Same question for location with specific activities:.
      ii. Java: sawah, Lahan PU, hutan rakyat, perhutani
      iii. Papua: timber harvesting, hunting
      iv. Kalimantan: (preliminary suggestions to be developed more after PM activity): oil palm, mining, community forest

6. Indicating various land uses on the map ask a series of questions on if the area of this land is currently increasing or decreasing. *This will be dependent on the land uses indicated in the Participatory Mapping exercise*.
   a. Do you think the area dedicated to kebun will increase or decrease? Why?
   b. Do you think the area dedicated to rice field will increase or decrease? Why?

7. In the past, have any villagers had difficulty accessing or using land? Explain how.

8. Do people in the village currently have difficulty accessing and using land? Explain.

9. Can villagers' children expect to have access to the same land and forest resources? Whether yes or no, ask why? If another time frame is mentioned this should be documented.

10. Do you foresee any obstacles to future land access? *Such as: actors that may also claim rights to this land, actors that may illegally encroach on your land, climate/environmental change, etc.*
    If yes, do you have a strategy to overcome this obstacle? *Is there anything you could do to avoid the obstacle?*

Village : Name of Respondent:
Date : Page : /

**Polygon** (*may be number or symbol*)	**1. User(s) and use**			**2. Controller(s)**	**3. Arrangement**	**4. Authority**
	People allowed and activity	People not allowed	Users that are unauthorized			
Example: 1	*Villagers: Access*	*Outsiders*		*Customary Leader*	*Ask for permission*	*Customary claim*

Annex 1 : Table for Questions 1 to 4

Village :
Name of Respondent:

Date :
Page : /

