# Supplementary material for: Considering land tenure in REDD+ participatory measurement, reporting, and verification: A case study from Indonesia
Source: PLoS One. 2017 Apr 13;12(4):e0167943. doi: 10.1371/journal.pone.0167943 (PMC5390967; doi:10.1371/journal.pone.0167943)
Supplement: S4 Fig — (PDF) [file pone.0167943.s004.pdf]

Desa : Nama FGD : Tanggal : Page : /

<table>
<tr><th colspan="10">Pemetaan Partisipatif</th></tr>
<tr><td rowspan="3">Penulis</td><td rowspan="3"></td><td rowspan="2" colspan="2">Pewawancara</td><td rowspan="2" colspan="2"></td><td>Dimasukkan oleh</td><td colspan="3"></td></tr>
<tr><td>Diperiksa oleh</td><td colspan="3"></td></tr>
<tr><td colspan="2">Asli atau Salinan</td><td>A</td><td>S</td><td>Nama file</td><td colspan="3"></td></tr>
<tr><td>Diperiksa oleh</td><td></td><td colspan="4">Ditulis di bagian belakang</td><td>Y</td><td>T</td><td>Sudah dicopy?</td><td></td></tr>
</table>

N°	Name	Job
1		
2		
3		
4		
5		
6		
7		
8		
9		
10		

Participatory mapping: correlation between land use (LU) and land cover (LC)

Material

9 maps: *5 base map in transparent (including road, river, settlement, any other relevant key features), 2 land cover (LC) in color, 2 Google Earth Pro*

GUIDELINE FOR MAPPING ACTIVITY

Acknowledgement: Methods between villages might be different, for instance in Java, we focus the mapping inside the village boundaries from BPS, whereas in Papua, we won't take in consideration any official boundaries, just the territory that villagers identified as their during COLUP project. However, in Java, we will ask if they have activities outside the boundary.

A- Familiarize people with the maps

1- Use only the high resolution Google Earth image. The high resolution image can be displayed in the village for the villagers to get familiarized with the map before the participatory activity.

2- Start the discussion about the BPS village boundary and identify if it is accurate or not and if there are mismatches (not necessary for Papua).

3- Identify the important locations, name of the different sub village units (dusun) or settlements, main road, river, water body…

B- Map Building

1- Villager's features and activities on a transparent base map

Drawing/updating the transparent base map (based on people's comments on the map familiarization): new village boundary (if necessary), key features (dusun, river, huts, roads, paths, harbor etc)

(If possible, draw as accurate as possible according to topography or natural elements. This will not be recognized officially because it is only based on one village's perception. The interest of drawing the boundary is to have the limit of the area of influence of villagers' activity upon the forest.)

Tasks:

a. Place a transparent base map over the high resolution map.

b. Draw any new features mentioned by local people on this base map (dusun, river, huts, roads, paths, harbor etc). It will be used as new base map.

c. Identify the activities of villagers on the new base map. We'll draw those information on the base map in the next step. The high resolution image can be used as reference to help the villagers to identify their activities.

2- <u>Land cover (LC) map based on villagers' perception and its correlation with our colored land cover map</u>

We already have the land cover high resolution and the land cover in color. However, this land cover map is derived from satellite imagery and the land cover classification is based on our visual interpretation of this image. In this activity, we need the villager's perception about their current land cover. We will use the high resolution image to help the villagers to see the features so they can define their own land cover.

<u>Important:</u> Do not use our colored land cover map before the villagers finished to draw their own, because it might influence their interpretation and the way they will draw their land cover. We will use this colored land cover map once the villagers finished to draw their own land cover.

Tasks:

a. Place a transparent new base map over the high resolution map.

b. Draw the land cover map based on the local people's perception (only with the help of the features on the new base map and the high resolution map).

c. Overlay the villagers' land cover map with our colored land cover map. Look where the overlaps and differences are between the two maps?

d. Do ground check where there are differences between villagers' land cover and our colored land cover. Please refer to the ground check section and GPS datasheet.

3- <u>Current land use (LU) map and its correlation with villagers' land cover (LC) map</u>

Explain that we are interested to draw the actual land use as accurate as possible and discuss on how to regroup the different land uses.

Tasks:

a. Place a transparent new base map over the high resolution map.

b. Ask the villagers' activities based on each land cover identified in the previous step by filling the column 2 & 3 in the table 1 in appendix 1 (LC description and villagers' activities by LC).

c. Regroup those activities by groups (we will call it **land use** (LU)) and find symbol for each group and fill column 4 & 5 in the table 1 in appendix 1.

d. Draw those land uses (LU) on a transparent new base map. The high resolution map can be used as reference to help the villagers to place those land uses.

e. Overlay the land use map with the villager's land cover map and see the correlation.

f. Overlay the land use map with our colored land cover map to fill the column 1 in table 1 in appendix 1. Look at the correlation.

4- <u>Past land cover (LC) and land use (LU) map (10 years time frame and more)</u>

Here there are 2 possibilities:

- The first one is that local people will not be able to draw the past land cover, in this case we ask for drawing where any change occurred.
- The second one is that local people are able to draw the past land cover.

In both cases, the idea is to refer to the current land cover so that we are able to identify what was the past LC or where any change occurred in the last 10 years and more. For that, we try to locate on the high resolution map where these change occurred and just mark or add symbol in that location.

Tasks:

a. Ask the historical event

b. Fill the table 2 in appendix 2.

At the same time:

c. Place a transparent base map over the high resolution map.

d. Draw the past land cover/use change based on the villager's perception. The high resolution image can be used here as a reference and to help them draw the land cover or land use.

e. Explain the changes (where, when, what for, how, by whom the changes occur?) (please refer to the table 2 appendix 2)

C- Ground check

1- Material

i. 2 GPS Garmin Etreck: to record the location/coordinate
ii. Data sheet (see table 4 in appendix 4): to write down the information about the location/coordinate
iii. Camera (personal/CIFOR): to take photo of north, south, east, west, and up to the location/coordinate
iv. Compass: to help knowing where is north, south, east, and west when taking the photos

2- Ground check activities

One day/one social scientist with the help of a local people (someone who knows better about the area). Walk around the village and take as many GPS point as possible for every LU in the LC.

3- Tasks

a. Go to the specific location where you want to take the coordinate
b. Wait until the GPS find a signal
c. Wait until you get an error accuracy less than 5 meters (if impossible to have less than 5 meters due to weather condition or any other circumstances, you can always record the point but try to have it as small as possible, say maximum 15 meters)
d. Record the location on your GPS
e. Record the information on the data sheet (see table 4 in appendix 4)
f. Use you compass to find out where is north
g. Take photos of north, south, east, west, and up
h. You can go to the next point and repeat the same processes
