# Supplementary material for: Considering land tenure in REDD+ participatory measurement, reporting, and verification: A case study from Indonesia
Source: PLoS One. 2017 Apr 13;12(4):e0167943. doi: 10.1371/journal.pone.0167943 (PMC5390967; doi:10.1371/journal.pone.0167943)
Supplement: S5 Fig — (PDF) [file pone.0167943.s005.pdf]

Desa : Nama FGD : Tanggal : Page : /

APPENDIX 1

Table 1. LC description and LU identification				
**LC Type (refer to LC map in color)**	**Villagers' LC description**	**Villagers' activities**	**LU Regrouping (Regroup all villagers' activities into similar group)[1]**	**Symbol (to link this table with the map)**

[1] For example, berburu burung, berburung buaya, berburu baby hutan can be grouped as "berburu"

Desa : Nama FGD : Tanggal : Page : /

APPENDIX 2

Table 2. Past land cover and land use (LC)

Historical events	Current land cover/land use	Past land cover/land use	Symbol to link with the LC map	When the change occurred?	What for, why, by whom are the changes?
**Example:**<br><br>**Expansion of the village**	**Settlement**<br><br>**Paddy field**	**Forest**<br><br>**Forest**	**xxx**<br><br>**ooo**	**1970**<br><br>**1970-1975**	**Build new house and expand the paddy field, to have more space to live in, by villagers themselves**
